# Wilms’ Tumor 1 (WT1): A Novel Immunomarker of Dermatofibrosarcoma Protuberans—An Immunohistochemical Study on a Series of 114 Cases of Bland-Looking Mesenchymal Spindle Cell Lesions of the Dermis/Subcutaneous Tissues

**DOI:** 10.3390/cancers13020252

**Published:** 2021-01-12

**Authors:** Eliana Piombino, Giuseppe Broggi, Mattia Barbareschi, Sergio Castorina, Rosalba Parenti, Giovanni Bartoloni, Lucia Salvatorelli, Gaetano Magro

**Affiliations:** 1 Department of Medical and Surgical Sciences and Advanced Technologies, G.F. Ingrassia, Azienda Ospedaliero-Universitaria “Policlinico Vittorio Emanuele”, Anatomic Pathology, School of Medicine, University of Catania, 95123 Catania, Italy; elianapiombino@hotmail.it (E.P.); giuseppe.broggi@phd.unict.it (G.B.); lucia.salvatorelli@unict.it (L.S.); 2 Pathology Unit, Department of Clinical Services, Santa Chiara Hospital, 38122 Trento, Italy; mattia.barbareschi@apss.tn.it; 3 Department of Medical and Surgical Sciences and Advanced Technologies, G.F. Ingrassia, Azienda Ospedaliero-Universitaria “Policlinico Vittorio Emanuele”, Anatomy, School of Medicine, University of Catania, 95123 Catania, Italy; sergio.castorina@unict.it; 4 Department of Biomedical and Biotechnological Sciences (BIOMETEC), Section of Physiology, University of Catania, 95123 Catania, Italy; parenti@unict.it; 5 Anatomic Pathology, A.R.N.A.S. Garibaldi Nesima, 95123 Catania, Italy; gbartolo58@gmail.com

**Keywords:** dermatofibrosarcoma protuberans, bland-looking spindle cell tumors, WT1, differential diagnosis, immunohistochemistry, immunomarker

## Abstract

**Simple Summary:**

Dermatofibrosarcoma protuberans (DFSP) is a superficial fibroblastic spindle cell sarcoma with a high rate of local recurrence (20% to 50%) but with a low metastatic potential. DFSP is characterized by *COL1A1-PDGFB* gene fusion and diffuse immunohistochemical expression of CD34. This immunomarker is especially useful in distinguishing DFSP from its morphological mimickers, especially when pathologists are faced with small biopsies. Apart from CD34, there are no additional diagnostic immunomarkers for DFSP, and thus, there is the need to identify more sensitive and specific markers for this sarcoma. Recently, Wilms’ tumor 1 (WT1) has been shown to be diffusely expressed in the cytoplasm of several benign and malignant mesenchymal spindle cell lesions. Based on this background, the aim of this study is to evaluate the immunohistochemical expression of WT1 protein in a series of bland-looking spindle cell lesions of the dermis/subcutis, emphasizing its potential diagnostic role in identifying DFSP among its morphological mimickers.

**Abstract:**

Purpose: to investigate the immunohistochemical expression and distribution of Wilms’ tumor 1 (WT1) (transcription factor produced by the tumor suppressor gene of the same name) in a series of 114 cases of bland-looking mesenchymal spindle cell lesions of the dermis/subcutaneous tissues to establish whether this immunomarker is differentially expressed in dermatofibrosarcoma protuberans (DFSP) versus its potential morphological mimickers. Methods: This retrospective multi-centric immunohistochemical study included 57 DFSP cases, 15 dermatofibromas, 5 deep fibrous histiocytomas, 8 neurofibromas, 5 spindle cell lipomas, 8 dermal scars, 6 nodular fasciitis, 5 cutaneous leiomyomas and 5 solitary fibrous tumors. Among the 57 DFSP cases, 11 were recurrent lesions; 2 non-recurrent cases exhibited an additional “*fibrosarcomatous*” overgrowth and 1 recurrent and 2 primary tumors contained a minority of “*giant cell fibroblastoma*” components. Results: Most DFSP (95% of cases) exhibited cytoplasmic staining for WT1; 11/11 residual/recurrent tumors showed diffuse and strong WT1 cytoplasmic immunoreactivity; apart from neurofibromas, WT1 expression was lacking in all the other cases studied. Conclusions: The cytoplasmic expression of WT1 may be exploitable as a complementary diagnostic immunomarker to CD34 in confirming the diagnosis of DFSP and to better evaluate the residual/recurrent tumor component.

## 1. Introduction

Dermatofibrosarcoma protuberans (DFSP) typically presents as a nodular subcutaneous mass with slow but persistent growth, often lasting several years. It is a relatively rare sarcoma, with an estimated prevalence of 1:10,000 and an incidence of about 1:200,000, generally occurring between the second and fifth decade of life. It typically occurs during adult life as a nodular mass of the skin. Although initially considered rare in children [1,2,3,4], an increasing number of cases have been reported even in childhood [2]. Males are more affected than females [2]. Although these tumors can occur in almost any location, they are most frequently found on the trunk and proximal extremities. Although it presents as a grossly well-circumscribed mass, this neoplasm usually infiltrates the dermis and subcutis. The tumor can reach the epidermis or spare an area of the dermis just below the epidermis (the grenz zone). The periphery of the tumor has a deceptively bland-looking appearance. This is particularly evident in the superficial areas, where the infiltration of the dermis by bland-looking cells can become a confounding factor in the differential diagnosis with dermatofibroma. In deeper regions, the tumor infiltrates along the connective septa and between the appendages and/or intertwines with lobules of subcutaneous adipose tissue, creating the characteristic “*honeycomb*” appearance. The central part of the tumor is composed of a uniform population of thin fibroblasts arranged into a predominant storiform growth pattern, with low vascularization. Mild nuclear atypia, low/moderate mitotic activity, as well as giant cells and inflammatory cells can be observed. Occasionally, DFSP may exhibit extensive myxoid stromal changes that can obscure the typical storiform growth pattern, closely mimicking a myxoid liposarcoma. Accordingly, the diagnosis of DFSP with abundant myxoid matrix usually requires the identification of typical areas, at least focally, within this context. There is increasing evidence that a small subgroup of DFSP contains areas indistinguishable from conventional fibrosarcoma (so-called “*dermatofibrosarcoma, fibrosarcomatous variant*”) and, more rarely, from undifferentiated pleomorphic sarcoma [5,6,7,8,9,10,11,12,13,14,15,16,17,18]. These areas, interpreted as a dedifferentiation tumor process, have a higher risk of local recurrence, while their metastatic potential is still debated. These tumors share the same general clinical features as normal DFSPs and, in most cases, fibrosarcomatous foci are already present in the primary tumor. Fibrosarcomatous foci of de-differentiation, which usually constitute up to 5–10% of the entire tumor, are characterized by “*herringbone”*—rather than storiform—or fasciculated architecture, being composed of spindle cells with moderate to focally severe nuclear atypia; mitotic activity is increased while immunoreactivity for CD34 is usually reduced when compared to the surrounding typical dermatofibrosarcoma areas. In addition, fibrosarcomatous areas are also characterized by a higher MIB-1 index and increased p53 positivity compared to the typical DFSP areas. Although a mitosis cut-off is not required to diagnose fibrosarcomatous de-differentiation, mitotic activity in these areas averages 7 to 15 mitosis/10 high power fields (HPF) compared to 1–3/10 HPF of traditional DFSP.

DFSP is characterized by immunohistochemical positivity for CD34 [5,6,7,8]. Although this antigen has been identified in a wide variety of soft-tissue neoplasms, its presence in DFSP suggests a close link with normal CD34+ dermal dendritic cells, including those surrounding the appendages, nerves, and vessels [5,7]. CD34 immunoreactivity has been shown to be useful in distinguishing DFSP from fibrohistiocytoma (dermatofibroma), especially when dealing with small biopsies, although cutaneous fibrohistiocytomas may sometimes show focal and weak expression of this immunomarker [9].

Making a correct diagnosis of DFSP is relatively straightforward if the tumor arises in the typical clinical setting and the classic morphological features are all recognizable. However, it is widely known that diagnostic problems may arise in that (i) similar morphological and/or immunohistochemical features may be shared by different neoplasms; (ii) considerable clinico-pathologic and immunohistochemical variability does exist; (iii) the diagnosis may be more challenging due to the increasing use of small biopsies in surgical practice. Accordingly, immunohistochemical analyses are currently mandatory as ancillary techniques in establishing the correct diagnosis. Over recent decades, only CD34 has been identified as a sensitive—not specific—marker for DFSP [5,6,7,8]. However, other neoplasms such as solitary fibrous tumor [19,20] and, occasionally, deep fibrous histiocytoma and neurofibroma [5,6,9] may show a similar immunoreactivity.

In recent years, several studies have demonstrated the presence of Wilms’ tumor 1 (WT1) protein within the cytoplasm in several benign and malignant tumors, suggesting its complex regulator activity in transcriptional/translational processes [21,22]. Although diffuse and strong WT1 cytoplasmic staining has been observed in several benign and malignant tumors, there is no information available about the expression of WT1 in DFPS. WT1 gene encodes a zinc-finger transcription factor, first identified as a tumor suppressor gene [21,23] playing a key role in Wilms’ tumor but also involved in proliferation and apoptosis, depending upon the cellular context [24,25,26]. The cellular localization of the WT1 protein has been a matter of debate over the last two decades. WT1 nuclear expression has been mainly observed by using antibodies directed against the C-terminal portion of the molecule (WT C-19 polyclonal antibody), while an exclusive cytoplasmic expression or coincident cytoplasmic and nuclear expression has been noticed with more recently generated available antibodies against the N-terminal portion (clone 6F-H2). In this regard, the cytoplasmic immunoreactivity was originally questioned and interpreted as non-specific staining due to cross-reactivities of the antibody or caused by formalin-fixation, as previously documented for other transcription factors such as c-myc gene product [27,28,29]. However, there is increasing evidence that the latter staining truly reflects the presence of the protein within the cytoplasm, suggesting its complex regulator activity in transcriptional/translational processes [30,31,32]. Recently, we showed that WT1 is also diffusely expressed in the cytoplasm of human fetal endothelial and skeletal muscle cells, as well as in developing sympathetic neuroblasts [33,34]. Interestingly, WT1 cytoplasmic immunostaining has also been documented in endothelial cells of most benign and malignant vascular tumors [35,36] in juvenile-type fibromatoses, infantile/congential fibrosarcomas, rhabdomyosarcomas, some neuroblastic tumors, some benign and malignant peripheral nerve sheath tumors, gastrointestinal stromal tumors (GIST), leiomyosarcomas, epithelioid cell myofibroblastomas of the breast, and in rhabdomyosarcoma [37,38,39], a tumor composed of malignant mesenchymal cells showing morphological, immunohistochemical, and ultrastructural features of skeletal muscle differentiation. These findings seem to support the hypothesis that not only nuclear but also WT1 cytoplasmic expression in some tumors recapitulates that observed during normal development [33,34]. Based on these findings obtained in both developmental and neoplastic tissues, it has been suggested that WT1 is a reliable marker of both endothelial and skeletal muscle differentiation [33,34].

The aim of this study is to investigate the immunohistochemical expression and distribution of WT1 (the transcription factor produced by the tumor suppressor gene of the same name) in a series of 114 cases of bland-looking mesenchymal spindle cell lesions of the dermis/subcutaneous tissues to evaluate its diagnostic utility in identifying DFSP.

## 2. Results

Immunohistochemical results, including staining distribution, extension, and intensity, have been summarized in Table 1. 

### 2.1. Clinical Data of the Cohort of Cases Included in the Study

The patients with DFSP were 30 males and 27 females with an age ranging from 20 to 77 years. Tumors occurred in the chest wall (*n* = 17 cases), shoulder (*n* = 6 cases), sub–clavicular region (*n* = 8 cases), scapula (*n* = 6 cases), mammary region (*n* = 6 cases), sovrapubic region (*n* = 2 cases), scalp (*n* = 3 cases), abdominal region (*n* = 4 cases), and arm (*n* = 5 cases). Patients with dermatofibroma were 10 females and 5 males with an age ranging from 9 to 84 years. Tumors occurred in the shoulder (*n* = 3 cases), arm (*n* = 4 cases), leg (*n* = 2 cases), scapula (*n* = 2 cases), ankle (*n* = 1 case), lumbar region (*n* = 1 case), and knee (*n* = 2 cases). Patients with deep fibrous histiocytoma were 3 males and 2 females with ages ranging from 29 to 67 years. Tumors occurred in the forearm (*n* = 2 cases), face (*n* = 1 case), gluteal region (*n* = 1 case), and supraclavicular region (*n* = 1 case). Patients with dermal scar were 5 females and 3 males with an age ranging from 21 to 76 years. Lesions occurred in the neck (*n* = 1 case), lumbar region (*n* = 1 case), leg (*n* = 2 cases), shoulder (*n* = 1 case), scalp (*n* = 2 cases), and mammary region (*n* = 1 case). Patients with spindle cell lipoma were 4 males and 1 female with an age ranging from 44 to 69 years. Tumors occurred in the cervical region (*n* = 3 cases) and shoulder (*n* = 2 cases). Patient with nodular fasciitis were 4 males and 2 females with an age ranging from 16 to 35 years. Tumors occurred in the chest wall (*n* = 2 cases), arm (*n* = 1 case), and leg (*n* = 3 cases). Patients with cutaneous leiomyomas were 3 males and 2 females with an age ranging from 41 to 55 years. Tumors occurred in the shoulder (*n* = 2 cases), leg (*n* = 1 case), and abdominal region (*n* = 2 cases). Patients with neurofibroma were 4 males and 4 females with an age ranging from 18 to 55 years. Tumors occurred in the arm (*n* = 2 cases), scalp (*n* = 1 case), abdominal region (*n* = 1 case), knee (*n* = 1 case), shoulder (*n* = 2 cases), and sub–clavicular region (*n* = 1 case). Patients with solitary fibrous tumor were 4 females and 1 male with an age ranging from 43 to 54 years. Tumors occurred in the subcutis of the wrist (*n* = 1 case), arm (*n* = 1 case), chest wall (*n* = 2 cases), and leg (*n* = 1 case).

### 2.2. Evaluation of Immunohistochemical Expression of WT1

WT1 immunostaining (both nuclear and cytoplasmic staining) was evaluated as follows: the percentage of positively stained cells was assessed by semi-quantitative optical analysis according to a four-tiered system (<1% positive cells = negative staining; 1–10% positive cells = focal staining; 11–50% positive cells = heterogeneous staining; >50% positive cells = diffuse staining). Staining intensity was graded into weak, moderate, or strong intensity.

### 2.3. DFSP

**Figure 1:**
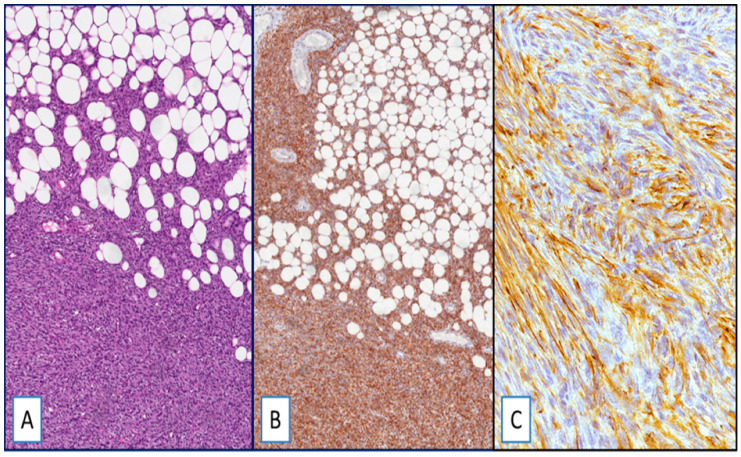
(**A**) Dermatofibrosarcoma protuberans (DFSP), classic-type: neoplastic cells infiltrate adipose tissue in a “honeycomb” pattern (hematoxylin and eosin (H&E); original magnification 100×); (**B**,**C**) strong and diffuse cytoplasmic staining for Wilms’ tumor 1 (WT1): (immunoperoxidase; original magnifications 100× (**B**) and 400× (**C**)).

**Figure 2:**
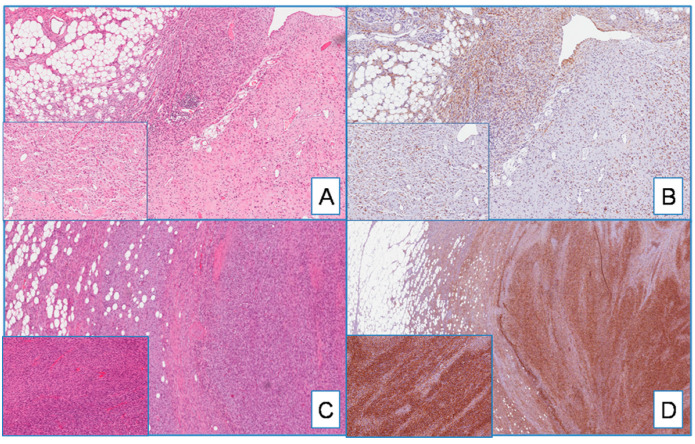
(**A**) DFSP with giant cell fibroblastoma component (on the right and insert) (H&E; original magnification 50×); (**B**) serial section showing diffuse cytoplasmatic staining for WT1 in both classic-type and giant cell components (insert) (immunoperoxidase; original magnification 50×); (**C**) DFSP with fibrosarcomatous component (on the right and insert) (H&E; original magnification 50×); (**D**) WT1 is diffusely and strongly expressed in both classic-type and fibrosarcomatous components (insert) (immunoperoxidase; original magnification 50×).

Most DFSP, namely 95% of cases (54 out of 57), exhibited cytoplasmic staining for WT1 (Figure 1). WT1-negative cases were represented by three primary, classic-type DFSPs. The immunohistochemical expression was diffuse, heterogeneous, or focal in 75%, 15%, and 6% of cases, respectively. With the exception of four cases (all classic-type primary DFSPs) showing a weak-to-moderate staining in different areas of the same tumor, the staining intensity was strong. Interestingly, the neoplastic cells of both the fibrosarcomatous and giant cell fibroblastoma components, found in two and three cases, respectively, were strongly and diffusely stained with WT1 (Figure 2).

**Figure 3:**
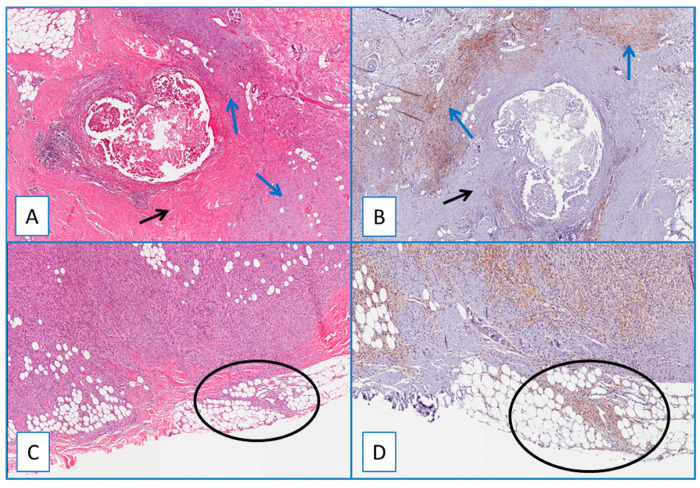
(**A**) Residual/recurrent DFSP: low magnification showing a residual neoplastic component (blue arrows) and fibroblasts/myofibroblasts of the surgically related scar tissue (black arrow) (H&E; original magnification 50×); (**B**) serial section showing a diffuse and strong cytoplasmic immunoreactivity of WT1 restricted to neoplastic cells (blue arrows), while the fibroblasts/myofibroblasts of the surgically related scar tissue are unstained (black arrow) (immunoperoxidase; original magnification 50×); (**C**) residual/recurrent DFSP: bland-looking neoplastic spindle cells infiltrating at the surgical margins (circle) (H&E; original magnification 50×); (**D**) serial section showing that these cells are stained with WT1, further supporting their neoplastic nature (circle) (immunoperoxidase; original magnification 50×).

A separate consideration is reserved for residual/recurrent DFSP. All residual/recurrent tumors showed diffuse and strong WT1 cytoplasmic immunoreactivity restricted to neoplastic cells, while the fibroblasts/myofibroblasts of the surgically related scar tissues were unstained (Figure 3). WT1 was detected in the cytoplasm of endothelial cells of intra- and extra-tumoral blood vessels, and this staining served as an internal control. Notably, no nuclear WT1 staining was obtained in all DFPS cases examined.

### 2.4. Non-DFSP Lesions

**Figure 4:**
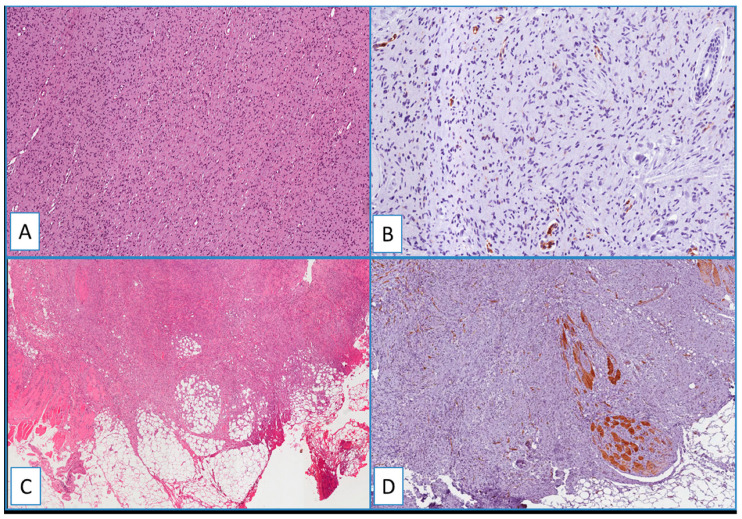
(**A**) Typical nodular dermal neurofibroma (H&E; original magnification 100×), showing a heterogeneous/focal and weak positivity for WT1; (**B**) (immunoperoxidase; original magnification 150×); (**C**) low magnification showing morphological details of a deep fibrous histiocytoma, containing foci of skeletal muscle infiltration (H&E; original magnification 50×); immunohistochemical analysis showed no detectable staining for WT1 in the neoplastic cells; (**D**) cytoplasmic staining of blood vessels and skeletal muscle cells (immunoperoxidase; original magnification 50×).

**Figure 5:**
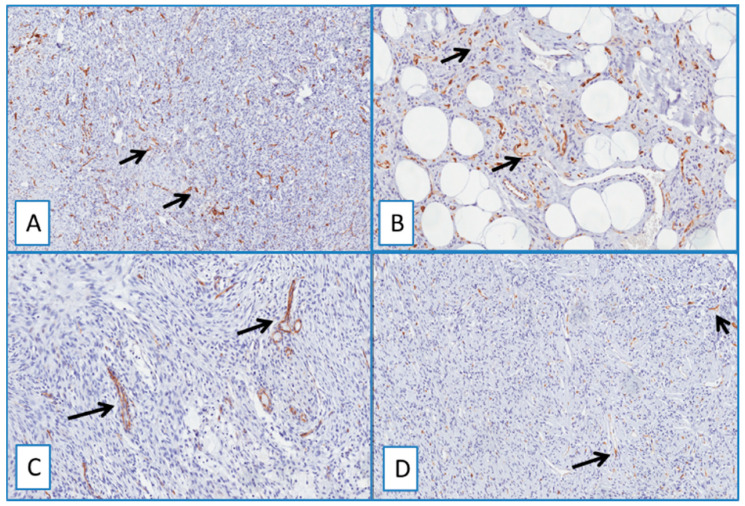
Immunohistochemical analyses showed no detectable staining for WT1 in dermatofibroma (**A**), spindle cell lipoma (**B**), nodular fasciitis (**C**), and solitary fibrous tumor (**D**). Cytoplasmic staining of blood vessels served as a positive internal control (arrows) (immunoperoxidase; original magnifications 50× (**A**,**D**) and 150× (**B**,**C**)).

Heterogeneous immunostaining was found in all cases of neurofibroma (Figure 4A,B). Notably, neither nuclear nor cytoplasmic staining was obtained in all the other cases included in this study, such as dermatofibroma, deep fibrous histiocytoma, spindle cell lipoma, solitary fibrous tumor, dermal scar, and cutaneous leiomyoma (Figure 4 and Figure 5).

WT1 was detected in the cytoplasm of endothelial cells of intra- and extra-tumoral blood vessels, and this staining served as an internal control.

## 3. Discussion

### 3.1. Diagnostic Utility of WT1 in Distinguishing DFSP from Its Morphological Mimickers

The diagnosis of soft-tissue tumors is one of the most difficult in the field of surgical pathology. Even more complex is the diagnosis of these lesions that arise primarily in the skin. This is due to the fact that many pathologists, despite a common expertise in the diagnosis of epithelial and melanocytic tumors, are often unfamiliar with soft tissue pathology. Although cutaneous mesenchymal lesions are composed of epithelioid and/or polygonal cells, most of them exhibit a proliferation of bland-looking spindle cells with a wide variety of growth patterns. Among these latter tumors, the most common neoplasm is certainly dermatofibroma, a benign tumor that, in most cases, is dermally centered. However, a tumor with partly overlapping morphological characteristics is DFSP, which, in addition to involving the dermis, extends/infiltrates into the subcutis [5,6]. Although in most cases, a distinction between dermatofibroma and DSFP is relatively straightforward, it should be admitted that in some circumstances, especially when a dermatofibroma infiltrates the subcutis, differential diagnostic problems may arise [5,6]. As cutaneous neurofibromas, both diffuse and nodular types, can also involve the dermis and subcutaneous tissue, they should be differentiated from dermatofibroma and DFSP. Although in recent years, several immunohistochemical markers have been identified as a diagnostic aid for several soft-tissue tumors, the same cannot be said for DFSP. In fact, the only reliable immunomarker for this neoplasm, to date, remains to be CD34, which, although highly sensitive, is not specific, as some cases of dermatofibromas and neurofibromas may be, at least focally, positive [5,6]. It is therefore necessary to identify new immunohistochemical markers for DSFP in order to avoid diagnostic errors with other neoplasms that represent morphological mimics. This is strongly supported by the fact that DFSP is capable of recurring locally and metastasizing in a small percentage of cases [5,6]. Our research group has recently shown that WT1 is a marker that can be expressed at the cytoplasmic level in childhood fibrosarcoma, juvenile-type fibromatoses, and rhabdomyosarcoma [33,34,35,36,37,38,39]. Therefore, we decided to test anti-WT1 antibodies, directed against its N-terminus portion, on a heterogeneous group of bland-looking spindle cell mesenchymal tumors of the dermis/subcutis, including DFSP. The results obtained are particularly interesting as only two tumor entities, namely DFSP and neurofibroma, resulted to be positive for WT1, with an expression restricted to the cytoplasm of the neoplastic cells. While the immunoreactivity for neurofibroma was rather heterogeneous in terms of extension and less strong in terms of staining intensity, DFSP exhibited a strong and widespread immunoreactivity in 70–90% of neoplastic cells. Of considerable interest was the absence of immunoreactivity, both cytoplasmic and nuclear, for WT1 in all the other neoplasms with which DFSP can be confused. Although differential diagnostic problems do exist between a diffuse neurofibroma infiltrating the dermis and DFSP, the co–expression of both CD34 and WT1 certainly does not help in their distinction. However, it should be remembered that neurofibroma, unlike DFSP, shows immunostaining, albeit with a variable extension, for S100 protein, a marker that is always negative in DFSP. Solitary fibrous tumor, which rarely arises in the dermo-hypodermic area, also expresses CD34, raising differential diagnostic problems with DFSP. However, the immunoreactivity for STAT-6, along with a concomitant negativity for WT1, favors the diagnosis of solitary fibrous tumor [19,20,40]. The present study shows for the first time that WT1 is a highly sensitive immunomarker for DFSP, suggesting its potential use in distinguishing this sarcoma especially from cellular dermatofibroma and deep fibrous histiocytoma.

### 3.2. Diagnostic Utility of WT1 in Recurrent/Residual DFSP

Apart from its sensitivity in identifying DFSP, WT1 is very helpful in distinguishing recurrent/residual tumor cells from fibroblasts/myofibroblasts of scar tissue after surgical excision, which can share CD34 immunostaining with the cells of DFSP. In this regard, we showed that in cases of residual/recurrent DFSP associated with surgically related scar tissue, WTI stained only the neoplastic cells, leaving unstained the fibroblastic/myofibroblastic cells of reactive scar.

As the use of small incisional biopsies is increasingly widespread in daily surgical practice, this clear-cut immunostaining difference is extremely helpful in distinguishing neoplastic from reactive spindled cells, thus providing essential clinical implications by improving the pathologist’s diagnostic accuracy in evaluating surgical margins and local recurrence of DFSP.

## 4. Materials and Methods

The cases were retrospectively retrieved from the surgical pathology archives of the section of Anatomic Pathology, G.F. Ingrassia Department of Medical, Surgical, and Advanced Technologies, at the University of Catania, from the Anatomic Pathology department of A.R.N.A.S. Garibaldi-Nesima of Catania, and from the and Anatomic Pathology department of Santa Chiara Hospital of Trento. Clinical data were obtained from the original pathology reports.

The following tumors were collected:

- Fifty-seven cases of DFSP; 11 of these cases were recurrent lesions; 2 primary cases exhibited an additional fibrosarcomatous overgrowth, while 2 primary and one recurrent tumor contained a minority of giant cell fibroblastoma component);
- Fifteen cases of dermatofibroma (classic type and cellular variants);
- Five cases of deep fibrous histiocytoma
- Eight cases of dermal scars;
- Five cases of spindle cell lipoma;
- Six cases of nodular fasciitis;
- Five cases of cutaneous leiomyomas;
- Eight cases of neurofibroma;
- Five cases of solitary fibrous tumor.

Hematoxylin and eosin (H&E)-stained slides and a variable number of slides stained with several antibodies were available for each case. All the H&E slides were reviewed by two surgical pathologists and the diagnoses were histologically confirmed using the current well-established morphologic criteria and immunohistochemical features.

Immunohistochemical analyses were performed as previously described [41], using the standard streptavidin–biotin labeling technique using the LSAB kit (Dako, Glostrup, Denmark) with appropriate positive and negative controls. Sections derived from paraffin-embedded specimens were deparaffinized in xylene for 15 min, rehydrated, and treated with 3% H_2_O_2_ for 10 min to block endogenous peroxidase activity, followed by extensive rinsing in double-distilled water and further rinsing for 15 min in 0.01 M phosphate-buffered saline (PBS), pH 7.4. Deparaffinized sections were incubated with anti-WT1 antibody (clone WT 6F-H2) (Dako, Glostrup, Denmark). Microwave pretreatment was crucial to enhance the staining in all samples examined. Accordingly, all sections were pretreated with citrate buffer (pH 6.0) and exposed to radiation in a microwave oven. To reduce the commonly seen non-specific immunoreactivity due to endogenous biotin, sections were pretreated with 10 mg/mL of ovalbumin in PBS followed by 0.2% biotin in PBS, each for 15 min at room temperature. Bound antibody was revealed by incubation with 3,3-diaminobenzidine (Sigma-Aldrich, St. Louis, MO, USA) in 0.01% H_2_O_2_ for 5 min at room temperature. Sections were then counterstained with hematoxylin, dehydrated, and mounted. Negative controls involved the omission of the primary antibody. The sections were examined with a Zeiss Axioplan light microscope (Carl Zeiss, Oberkochen, Germany) and photographed with the Aperio Scanscope CS2 system.

## 5. Conclusions

The present study suggests that cytoplasmic expression of WT1 is of complementary diagnostic value to CD34 in confirming the diagnosis of DFSP and to better define the presence of residual/recurrent tumors, especially when evaluating surgical excision margins. Accordingly, we suggest to include WT1 in the list of the antibodies panel when approaching the diagnosis of bland-looking spindle cell lesions of the dermis/subcutis.

## Figures and Tables

**Table 1:** Summary of the immunohistochemical findings.

Diagnosis/Number of Cases	Positive Cases (%)	Staining Extension Cases (%)	Staining Intensity Cases (%)
Dermatofibrosarcoma protuberans (*n* = 57)	54/57 (95%)	Diffuse: 42/57 (75%) Heterogeneous: 9/57 (15%) Focal: 6/57 (6%)	Strong: 53/57 (93%) Weak: 4/57 (7%)
Dermatofibroma (*n* = 15)	0/15 (0%)	No staining	No staining
Deep fibrous histiocytoma (*n* = 5)	0/5 (0%)	No staining	No staining
Dermal scars (*n* = 8)	0/8 (0%)	No staining	No staining
Spindle cell lipoma (*n* = 5)	0/5 (0%)	No staining	No staining
Nodular fasciitis (*n* = 6)	0/6 (0%)	No staining	No staining
Cutaneous leiomyomas (*n* = 5)	0/5 (0%)	No staining	No staining
Neurofibroma (*n* = 8)	8/8 (100%)	Heterogeneous 8/8 (100%)	Weak/moderate 8/8 (100%)
Solitary fibrous tumor (*n* =5)	0/5 (0%)	No staining	No staining

## Data Availability

The data presented in this study are available in the surgical pathology archives of the section of Anatomic Pathology, G.F. Ingrassia Department of Medical, Surgical, and Advanced Technologies, at the University of Catania.
